# Disulfidptosis: A new type of cell death

**DOI:** 10.1007/s10495-024-01989-8

**Published:** 2024-06-17

**Authors:** Fei Xiao, Hui-Li Li, Bei Yang, Hao Che, Fei Xu, Gang Li, Cheng-Hui Zhou, Sheng Wang

**Affiliations:** 1 Department of Anesthesiology, Beijing Anzhen Hospital, Capital Medical University, Beijing, China; 2 Pediatric Cardiac Center, Beijing Anzhen Hospital, Capital Medical University, Beijing Institute of Heart Lung and Blood Vessel Diseases, Beijing, China; 3 Department of Emergency, The State Key Laboratory for Complex, Severe and Rare Diseases, Peking Union Medical College Hospital, Chinese Academy of Medical Science and Peking Union Medical College, Beijing, China; 4 Linzhi People’s Hospital, Linzhi, Tibet China

**Keywords:** SLC7A11, NADPH, Glucose starvation, Cystine, Disulfide bond, Disulfidptosis

## Abstract

Disulfidptosis is a novel form of cell death that is distinguishable from established programmed cell death pathways such as apoptosis, pyroptosis, autophagy, ferroptosis, and oxeiptosis. This process is characterized by the rapid depletion of nicotinamide adenine dinucleotide phosphate (NADPH) in cells and high expression of solute carrier family 7 member 11 (SLC7A11) during glucose starvation, resulting in abnormal cystine accumulation, which subsequently induces andabnormal disulfide bond formation in actin cytoskeleton proteins, culminating in actin network collapse and disulfidptosis. This review aimed to summarize the underlying mechanisms, influencing factors, comparisons with traditional cell death pathways, associations with related diseases, application prospects, and future research directions related to disulfidptosis.

## Introduction

Cells are the basic organizational units of life. Therefore, cell proliferation, differentiation, and death play important roles in different stages of life. Cell death is a fundamental physiological process in organisms and is crucial for maintaining the stability of the internal environment [1]. In recent years, disulfidptosis, a newly identified form of cell death, has garnered increasing attention. On February 6, 2023, the research group led by Professors Boyi Gan and Junjie Chen published their findings in Nature Cell Biology, unveiling the mechanism of disulfide stress-induced cell death and naming this new mode of cell death disulfidptosis [2].

Although the mechanism of disulfidptosis has not been fully elucidated, studies indicate that increased SLC7A11 protein expression is a critical factor in the occurrence of disulfidptosis. SLC7A11 is a transporter protein that is responsible for translocating cystine from the outside of the cell to the inside. Under conditions of glucose deprivation, increased expression of SLC7A11 results in substantial cystine accumulation, subsequently inducing disulfide stress and leading to cell death [2–4].

Recent studies have suggested that disulfidptosis plays a significant role in the onset and progression of various diseases. Associations have been observed with cancer [2, 5, 6], neurodegenerative diseases [7, 8], cardiovascular diseases [9], and liver diseases [10–12], and other conditions are closely related to disulfidptosis [13–15].

Consequently, comprehensive research on its mechanism holds significant clinical relevance, offering insights into the fundamental nature and principles of life and suggesting novel approaches for disease prevention and treatment.

## Differences and relationships between disulfidptosis and traditional cell death modes

Disulfidptosis is a novel form of cell death that is distinguished from traditional cell death modes by its unique mechanism, morphological characteristics, and regulatory networks. Disulfidptosis primarily results from NADPH depletion in cells expressing high levels of SLC7A11 under conditions of glucose deprivation, leading to the abnormal accumulation of cystine and other disulfides and culminating in disulfide stress and rapid cell death [2, 16]. Morphologically, disulfidptosis is characterized by an increase in disulfide bond levels within the cytoskeleton, leading to actin filament contraction, disruption of cytoskeletal integrity, and consequent cell death [2].

The traditional modes of cell death include apoptosis, pyroptosis, autophagy, ferroptosis, and oxeiptosis. Programmed processes are triggered by specific signaling pathways and regulatory networks. For instance, apoptosis caspase activation [17–19], pyroptosis is initiated by specific signaling pathways involved in inflammatory responses [20–23], and autophagy is initiated by autophagosome formation [24–26].

Although disulfidptosis and traditional cell death are distinct, they are not entirely separate. Interactions and connections likely exist between them. For example, disulfidptosis and ferroptosis are connected (Table 1). SLC7A11 is a specific cysteine transporter and a key regulatory protein associated with ferroptosis and disulfidptosis [2]. Downregulation of SLC7A11 indirectly inhibits the activity of glutathione peroxidase 4 (GPX4) by suppressing the cysteine metabolic pathway, leading to reduced intracellular cysteine levels and the depletion of glutathione (GSH) biosynthesis, which in turn leads to the accumulation of lipid peroxides and ultimately induces cell death by ferroptosis [27] and inhibits the occurrence of disulfidptosis [2, 3]. Upregulating SLC7A11 can indirectly promote the activity of GPX4 by promoting the cysteine metabolic pathway, leading to increased intracellular cysteine levels and GSH biosynthesis, which in turn inhibits cell death by ferroptosis. If cystine accumulates at this time, disulfidptosis may occur [13].

**Table 1:** Core molecular mechanisms of various cell death modes

Cell death modalities	Date	The core molecular mechanisms	Reference(s)
Disulfidptosis	2023	When the NADPH supply is limited by glucose deprivation conditions, high cystine uptake in cells with high SLC7A11 expression results in intracellular NADPH depletion, the excessive accumulation of cystine and other disulfide molecules, and abnormal disulfide bond formation in actin cytoskeleton proteins, culminating in actin network collapse and disulfidptosis. (Fig. 1)	[2, 4, 6, 9, 16, 28–32]
Alkaliptosis	2018	The molecular mechanisms underlying alkaliptosis involve pH-induced alkalization, mainly through the activation of JTC801, and the downregulation of carbonic anhydrase 9 (CA9), which depends on the IKBKB-NF-κB pathway, can induce the occurrence of alkaliptosis. (Fig. 2)	[32–34]
Oxeiptosis	2018	Oxeiptosis is a novel type of caspase-independent cell death that is initiated by oxygen radicals and is regulated by the KEAP1-PGAM5-AIFM1 pathway. (Fig. 3)	[32, 35, 36]
Autophagy	2013	Autophagy is a widespread degradation/recycling system in eukaryotic cells that uses the lysosomal system to degrade damaged long-lived proteins and organelles into small molecules such as amino acids, nucleotides, and free fatty acids that can be reused, providing cells with raw materials and energy for the synthesis of new proteins and organelles. (Fig. 4)	[32, 37–42]
Ferroptosis	2012	Due to ferrous iron and lipoxygenase, unsaturated fatty acids on the cell membrane undergo lipid peroxidation, concomitant with the downregulation of the intracellular antioxidant system. This confluence of events collectively promotes ferroptosis. (Fig. 5)	[27, 31, 32, 40, 41, 43–47]
Parthanatos	2009	Activation of NMDA receptors stimulates nNOS-mediated generation of ONOO^−^, this factor and ROS damage DNA strands, thereby activating PARP-1. When the DNA damage is high, the excessive activation of PARP-1 leads to abundant PAR polymer formation in the nucleus; some of the PARy lated carrier proteins exit the nucleus and cause the release of AIF from a pool on the outer mitochondrial membrane. Once in the cytosol, AIF can bind to MIF. Together, they enter the nucleus and produce large-scale DNA degradation and cell death. (Fig. 6)	[31, 32, 40, 45–51]
Entosis	2007	Entotic cell death is a form of "cannibalism" in which phagocytic cells engulf target cells through entosis, and then under the action of lysosomes, target cells within the entotic vesicles are gradually decomposed into small fragments, which are released into the cytoplasm, ultimately leading to the death of the target cells. Cell adhesion and cytoskeleton rearrangement pathways (such as actin, myosin, RHOA, and ROCK) play important roles in controlling the induction of entosis. In addition to cell adhesion and cytoskeleton rearrangement pathways, other signaling molecules and regulatory factors (such as CDC42) participate in the regulation of entosis through different mechanisms	[32, 40, 42, 52]
Necroptosis	2005	FASL, TRAIL, TNF and IFN-1 activates its receptor, and MLKL, RIPK1 and RIPK3 are recruited to assemble the necrosome through phosphorylation. The phosphorylation-mediated activation of MLKL and subsequent MLKL-mediated membrane pore formation results in necroptosis. In response to TNF-α induced necroptosis, PGAM5 is recruited to the RIPK1/RIPK3 complex on the outer mitochondrial membrane, where it triggers Drp1-mediated mitochondrial fragmentation, which is considered an obligatory step in necroptosis. (Fig. 7)	[31, 32, 40–42, 45, 51, 53]
NETosis	2004	NETosis is a form of cell death driven by NETs that mainly involves NADPH oxidase-mediated ROS production and histone citrullination. These processes eventually lead to chromatin decondensation, nuclear membrane destruction, and the release of chromatin fibers. ROS production and histone citrullination are key regulatory factors in NETosis. (Fig. 8)	[32, 40, 47, 54]
Pyroptosis	2001	Pyroptosis is a form of programmed cell death triggered by inflammasomes that mainly depends on the activation of caspase. Activated caspase cleaves the GSDM protein and releases its N-terminal domain, which binds membrane lipids and perforates the cell membrane, resulting in changes in cell osmotic pressure. The cell then expands until the cell membrane bursts, resulting in scorched cells. (Fig. 9)	[31, 32, 40, 41, 45, 46, 51, 55–59]
Lysosome-dependent cell death	2000	Lysosome-dependent cell death is triggered by ROS or lipid metabolites, and an increase in ROS is one of the main triggers of the increase in calcium, which can occur through the hyperactivation of TRPM2, calcium efflux from lysosomes, leading to LMP, and the release of cathepsins into cytosol. Cathepsins catalyze multiple substrates, including Bid and apoptotic proteins and initiate caspase-dependent cell death. In addition, ER stress can induce cytosolic calcium increase. High cytosolic calcium stimulates the activation of calpain, leading to the degradation of lysosomal membrane proteins such as LAMP1/2, causing lysosomes to rupture and resulting in lysosome-dependent cell death. (Fig. 10)	[32, 40, 45, 47, 60]
Apoptosis	1972	Apoptosis is an active and orderly cell death process determined by genes. When cells encounter internal and external environmental factors, suicide protection measures regulated by genes are initiated to remove nonessential cells or cells that are about to undergo specialization in the body. During this process, cells are shed from the body or lyse to form several apoptotic bodies, which are quickly cleared by macrophages or neighboring cells. Apoptosis is divided into exogenous apoptosis and endogenous apoptosis. It mainly includes the death receptor pathway and the mitochondrial pathway. The death receptor pathway is initiated by the binding of death receptors on the cell surface to ligands, while the mitochondrial pathway activates apoptotic enzymes by releasing proapoptotic factors such as cytochrome C from mitochondria	[32, 40, 42, 45, 61]

**Fig. 1:**
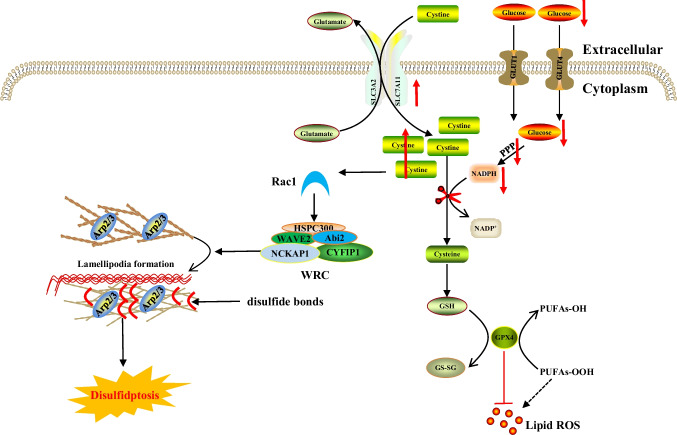
The core molecular mechanisms of disulfidptosis. When the NADPH supply is limited under glucose deprivation conditions, high cystine uptake by cells with high SLC7A11 expression results in intracellular NADPH depletion, the excessive accumulation of cystine and other disulfide molecules, and abnormal disulfide bond formation in actin cytoskeleton proteins, culminating in actin network collapse and disulfidptosis. Rac1-WRC-mediated branched actin polymerization and lamellipodia formation likely provide supporting conditions for disulfide bond formation in actin cytoskeleton proteins, thereby facilitating disulfidptosis. Abbreviations: SLC7A11, solute carrier family 7 member 11; SLC3A2, solute carrier family 3 member 2; GLUT1/4, glucose transporter 1/4; PPP, pentose phosphate pathway; NADPH, nicotinamide adenine dinucleotide phosphate; GSH, glutathione; GSSG, oxidized glutathione; GPX4, glutathione peroxidase 4; Arp2/3, actin-related protein 2/3 complex; RAC1, RAS-related C3 botulinum toxin substrate 1; WRC, WAVE regulatory complex; HSPC300, hematopoietic stem/progenitor cell protein 300; NCKAP1, NCK-associated protein 1; CYFIP1, cytoplasmic FMR1-interacting protein 1

**Fig. 2:**
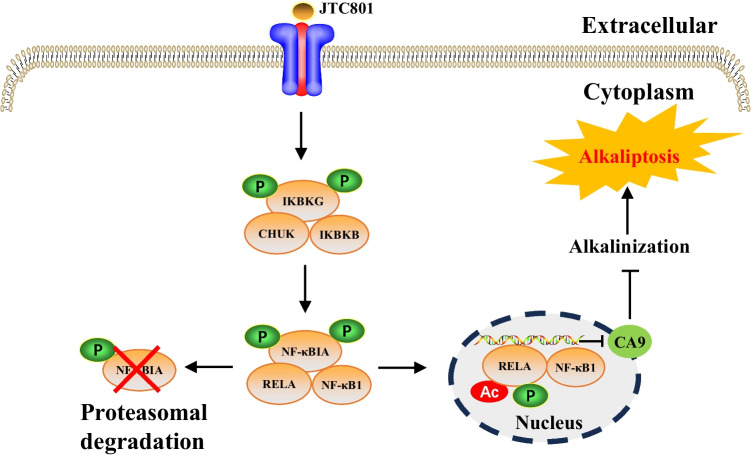
The core molecular mechanisms of alkaliptosis. The molecular mechanisms underlying alkaliptosis involve pH-induced alkalization, mainly through the activation of JTC801, and the downregulation of carbonic anhydrase 9 (CA9), which is dependent on the IKBKB-NF-κB pathway and can induce alkaliptosis. Abbreviations: IKBKB, inhibitor of nuclear factor κB kinase subunit-β; CHUK, component of inhibitor of nuclear factor kappa B kinase complex; IKBKG, inhibitor of nuclear factor kappa B kinase regulatory subunit gamma; NF-κBIA, NF-κB inhibitor alpha; RELA, RELA proto-oncogene, NF-κB subunit; NF-κB1, nuclear factor kappa B subunit 1; CA9, carbonic anhydrase 9

**Fig. 3:**
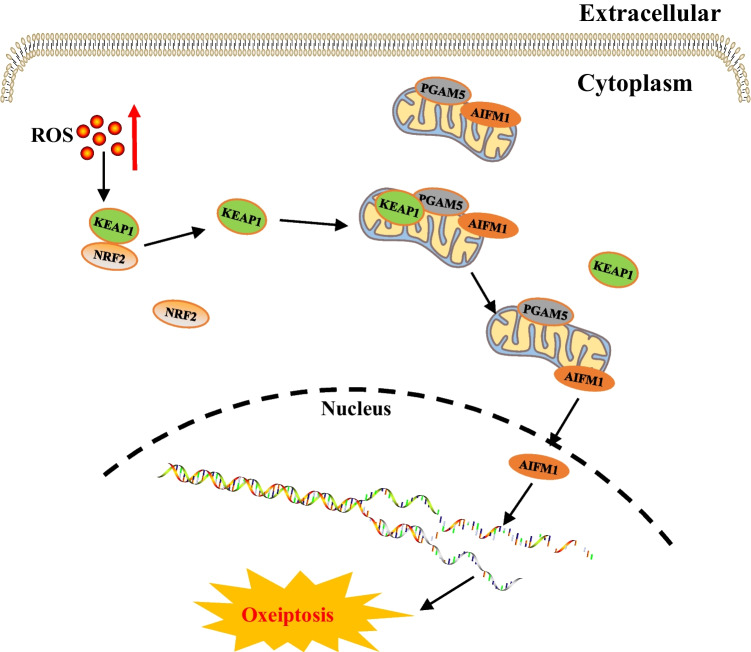
The core molecular mechanisms of oxeiptosis. High intracellular ROS levels induce conformational changes in KEAP1, triggering its dissociation from NRF2. KEAP1 translocates into mitochondria and mediates the release of AIFM1 from PGAM5 and AIFM1 translocation to the nucleus, where it dephosphorylates AIFM1 at S116, triggering cell death. Abbreviations: KEAP1, Kelch-like ECH-associated protein-1; NRF2, Nuclear factor erythroid 2-related factor 2; PGAM5, phosphoglycerate mutase family 5; AIFMI, apoptosis-inducing factor mitochondria associated 1

**Fig. 4:**
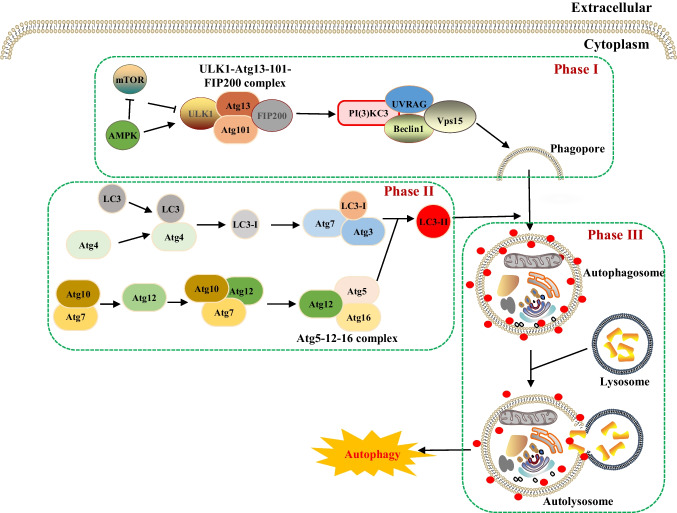
The core molecular mechanisms of autophagy. Autophagy can be divided into three stages: the induction stage, the formation stage of autophagosomes, and the formation of autophagic lysosomes and the degradation of their contents. 1. Autophagy induction stage: The ULK1-Atg13-Atg101-FIP200 complex transmits autophagy signals to the nucleus, and the Class III PI3K-Beclin-1 complex induces the formation of a double-layer membrane, leading to the accumulation of phagocytic vesicles. 2. Formation of the autophagosome: On the one hand, Atg7 and Atg10 activate and transport Atg12, which in turn binds to Atg5 and Atg16 to form the Atg5-Atg12-Atg16 complex. On the other hand, LC3 is decomposed by Atg4 to form LC3-I, which is activated by Atg7 and Atg3 and accumulates on the autophagosome membrane under the induction of the Atg5-Atg12-Atg16 complex to form LC3-II. The membrane extends and surrounds the intracellular degradation substrate to form an autophagosome. 3. Formation of autophagosomes and degradation of their contents: Through the interaction of LAMP-1, LAMP-2, GTPase-RAB-7, and other proteins, autophagosomes fuse with lysosomes to form autophagosomes, which release hydrolytic enzymes to fully degrade substrates. Abbreviations: Atg, Autophagy-related gene; LC3, microtubule-associated protein light-chain 3; PI3K, phosphoinositide 3-kinase; LAMP, lysosome-associated membrane protein; GTPase, GTP hydrolase; RAB-7, ras-related protein Rab-7

**Fig. 5:**
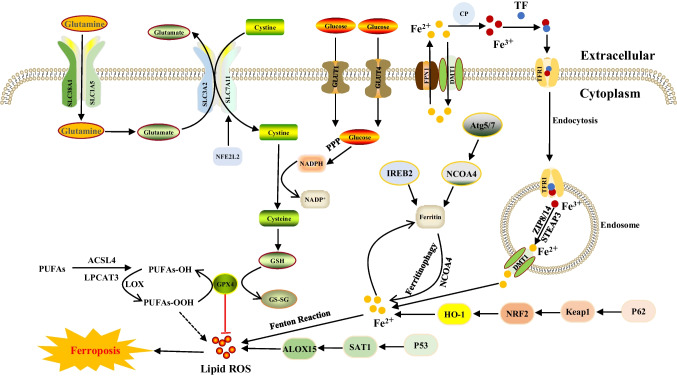
The core molecular mechanisms of ferroptosis. PUFAs are oxidized in a stepwise manner into lipid hydroperoxides (PUFAs-OH) by ACSL4, LPCAT3 and PUFA-OOH by LOX. GPX4 uses GSH as a substrate to catalyze the transformation of lipid hydroperoxides into hydroxy derivatives, limiting lipid peroxidation. System Xc- inhibition causes GSH depletion and attenuates GPX4 activity, leading to lipid peroxidation and ferroptosis. Moreover, iron overload generates hydroxyl radicals via the Fenton reaction, which also contributes to lipid peroxidation and ferroptosis. Abbreviations: SLC38A1, solute carrier family 38 member 1; SLC1A5, solute carrier family 1 member 5; SLC3A2, solute carrier family 3 member 2; SLC7A11, solute carrier family 7 member 11; Glucose Transporter 1/4; FPN1, ferroportin-1; DMT1, divalent metal transporter 1; TF, transferrin; CP, ceruloplasmin; TFR1, transferrin receptor 1; NFE2L2, nuclear factor erythroid 2-related factor 2; PPP, pentose phosphate pathway; NADPH, nicotinamide adenine dinucleotide phosphate; GSH, glutathione; GPX4, glutathione peroxidase 4; GSSG, oxidized glutathione; ALOX15, arachidonic acid 15-lipoxygenase; SAT1, spermidine/spermine N1-acetyltransferase 1; P53, tumor protein 53; HO-1, heme oxygenase-1; NRF2, nuclear factor erythroid 2-related factor 2; Keap1, kelch-1ike ECH-associated protein 1; P62, prostacyclin; NCOA4, nuclear receptor coactivator 4; IREB2, iron-responsive element binding protein 2; Atg5/7, autophagy related 5/7

**Fig. 6:**
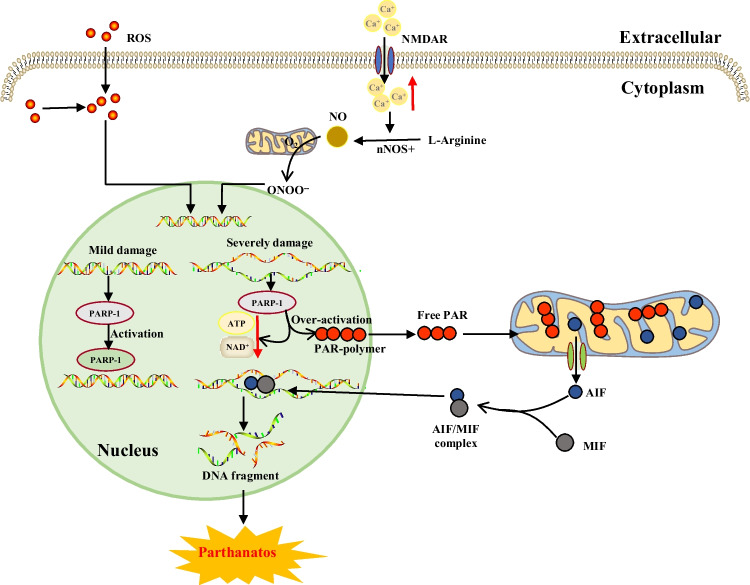
The core molecular mechanisms of parthanatos. The activation of NMDA receptors stimulates NO synthase (nNOS). The abundant levels of NO and superoxide spontaneously generate peroxynitrite (ONOO^−^). Along with other ROS, this strong prooxidant damages DNA strands and thereby causes the activation of PARP-1. When DNA damage is high, the consequent overactivation of PARP-1 leads to abundant PAR polymer formation in the nucleus; some of the PARy lated carrier proteins exit the nucleus and cause the release of AIF from a pool on the outer mitochondrial membrane. Once in the cytosol, AIF can bind to MIF. Together, these proteins enter the nucleus and cause large-scale DNA degradation and cell death. Abbreviations: ROS, reactive oxygen species; NO, nitric oxide; NMDA, N-methyl-D-aspartic acid receptor; nNOS, nitric oxide synthase; ONOO^−^, peroxynitrite; PARP-1, poly-ADP-ribosome-polymerase 1; NAD^+^, nicotinamide adenine dinucleotide; PAR, poly-ADP-ribose; AIF, apoptosis-inducing factor; MIF, macrophage migration inhibitory factor

**Fig. 7:**
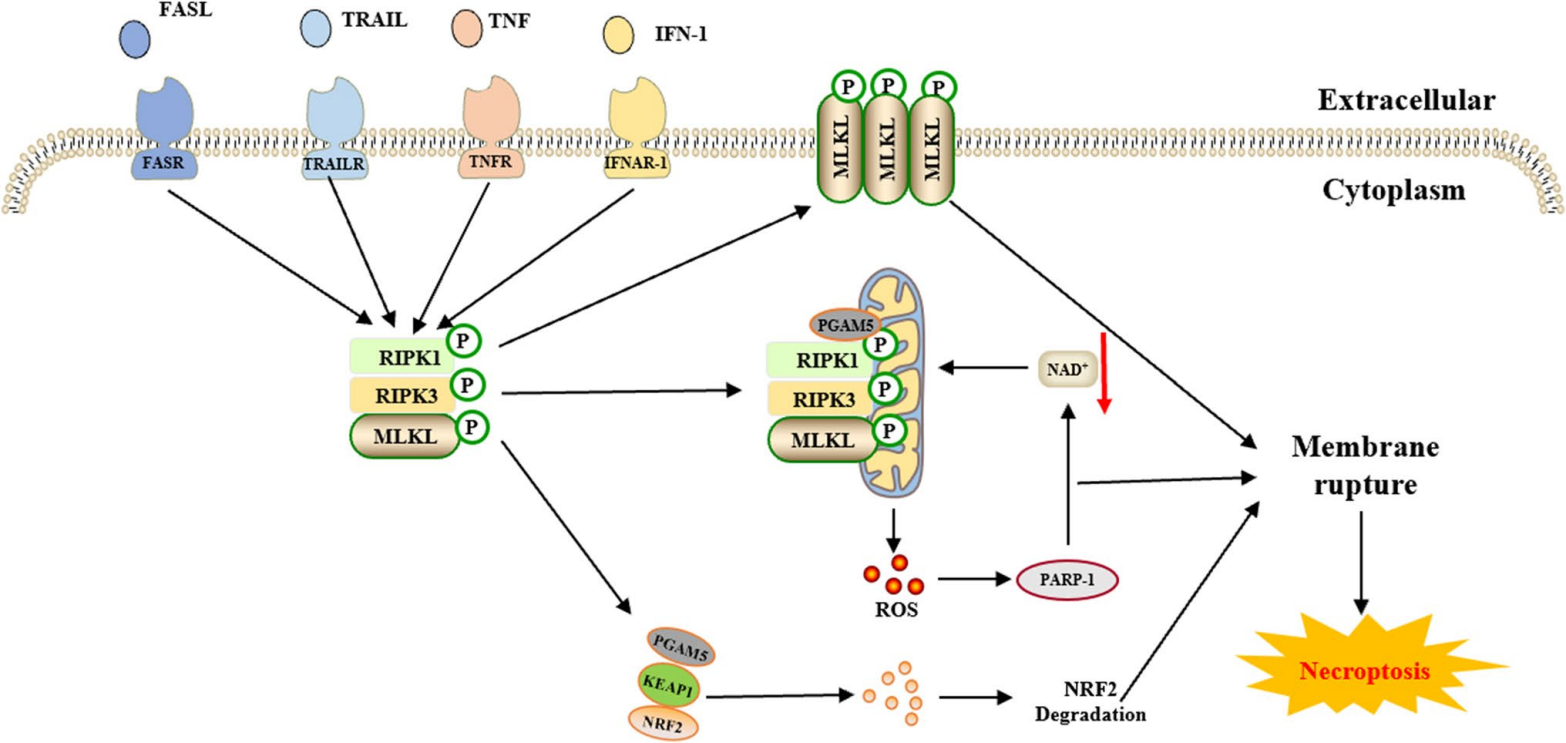
The core molecular mechanisms of necroptosis. FASL, TRAIL, TNF and IFN-1 activate each of their receptors, and MLKL, RIPK1 and RIPK3 are recruited to assemble the necrosome through phosphorylation. Phosphorylation-mediated activation of MLKL and subsequent MLKL-mediated membrane pore formation result in necroptosis. In response to TNF-α-induced necroptosis, PGAM5 is recruited to the RIPK1/RIPK3 complex on the outer mitochondrial membrane, where it triggers Drp1-mediated mitochondrial fragmentation and the release of large amounts of ROS, thereby activating PARP-1 and resulting in a decrease in NAD^+^ production and subsequent cycling, which is considered an obligatory step in necroptosis. Abbreviations: FASL, factor-related apoptosis ligand; TRAIL, TNF-related apoptosis-inducing ligand; TNF, tumor necrosis factor; IFN-1, interferon-1; MLKL, mixed lineage kinase domain-like protein; RIPK1, receptor-interacting protein kinase 1; RIPK3, receptor-interacting protein kinase 3; PGAM5, phosphoglycerate mutase family 5; KEAP1, Kelch-like ECH-associated protein 1; NRF2, nuclear factor erythroid 2 related factor 2; PARP-Q, poly-ADP-ribose polymerase; NAD^+^, nicotinamide adenine dinucleotide

**Fig. 8:**
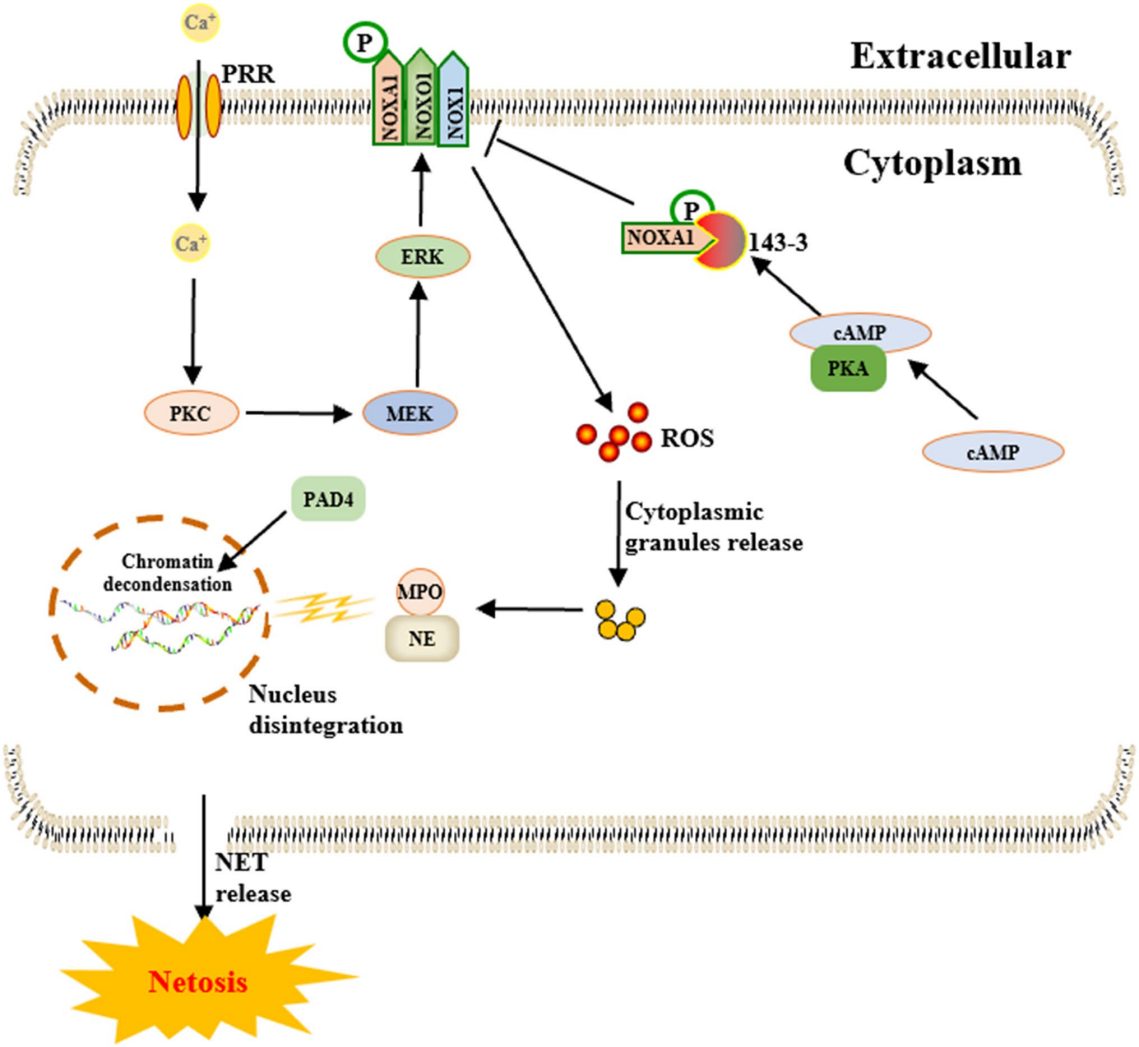
The core molecular mechanisms of NETosis. NETosis is initiated by the activation of neutrophils via PRRs and the subsequent influx of Ca^2+^. This triggers a cascade of events involving Ca^2+−^dependent PKC, the PKC-MEK-ERK pathway, and NOX phosphorylation, leading to ROS production. Excessive ROS cause cytoplasmic granule degradation and the release of NE, MPO and PAD4 to the nucleus, leading to chromatin decondensation and ultimately resulting in cell rupture and NET release. PKA-mediated phosphorylation of NOXA1 recruits 14–3-3 proteins, which block the assembly of the NOX1 holoenzyme, ultimately preventing ROS production. Moreover, supraphysiological cAMP concentrations inhibit the formation of NETs and ROS bursts. These findings highlight the role of cAMP signaling in inhibiting NETosis via PKA. Abbreviations: PRRs, pattern recognition receptors; PKC, protein kinase C; MEK, mitogen-activated extracellular signal-regulated kinase; ERK, extracellular regulated protein kinase; NADPH, nicotinamide adenine dinucleotide phosphate; NOX, NADPH oxidase; NE, neutrophil elastase; MPO, myeloperoxidase; PAD4, peptidyl arginine deiminase 4; NETs, neutrophil extracellular traps; PKA, protein kinase A; NOXA1, NADPH oxidase activator 1; NOX1, NADPH oxidase 1; cAMP, cyclic adenosine monophosphate

**Fig. 9:**
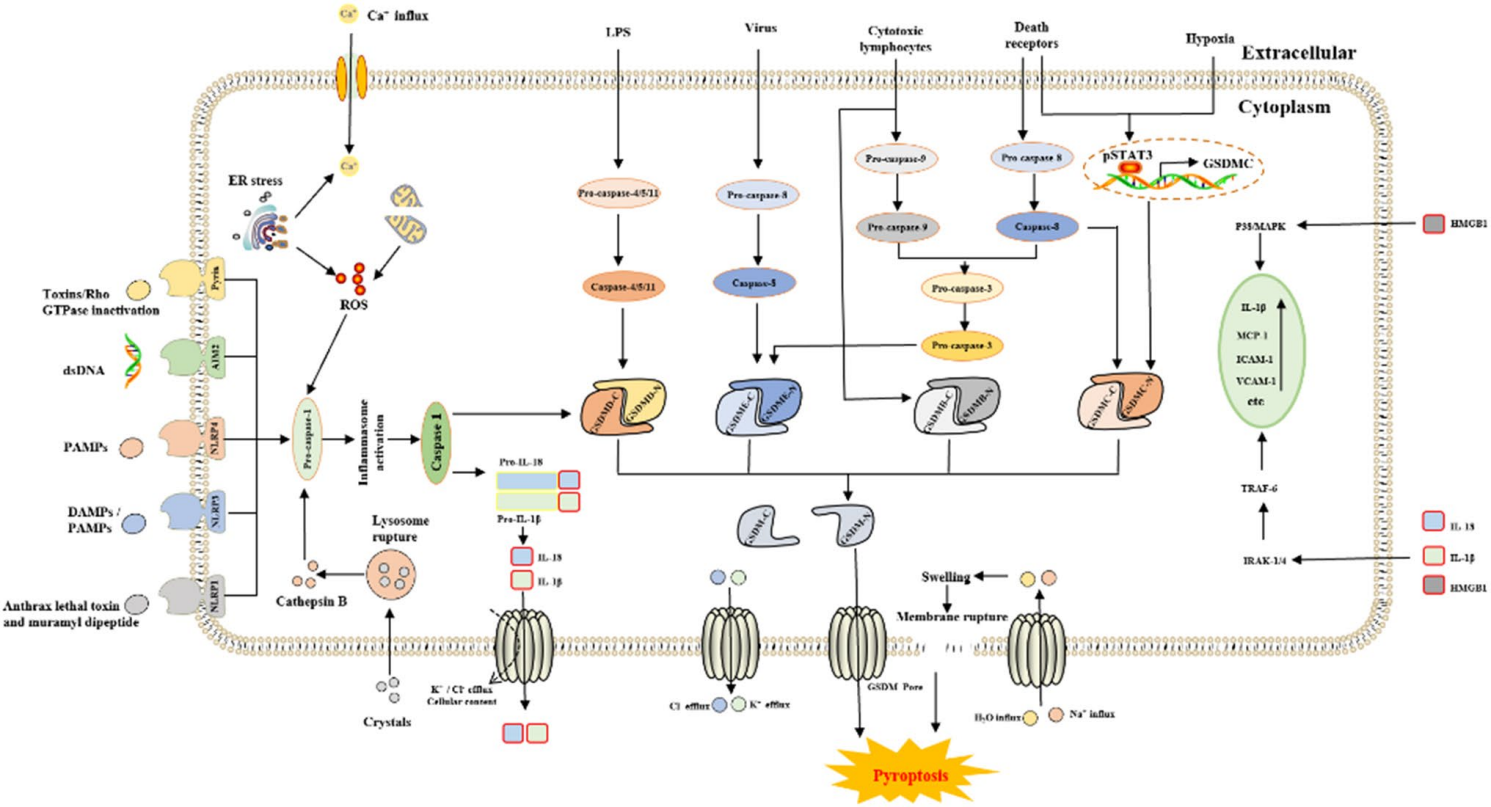
The core molecular mechanisms of pyroptosis. In response to DAMPs and PAMPs, cytosolic canonical inflammasomes (NLRP3, NLRP1, NLRC4, AIM2, pyrin, etc.) can respond to microbial infection (microbial toxins, etc.) or danger signals (dsDNA and crystals, etc.) to activate caspase-1, while noncanonical inflammasomes directly respond to LPS or other stimuli to activate caspase-4/5/11. After the activation of inflammatory caspases, pro-IL-1β, pro-IL-18, and GSDMD are cleaved to liberate N-terminal GSDMD (GSDMD-N), which forms pores on the plasma membrane and releases inflammatory mediators (IL-1β, IL-18, etc.). Other pathways involved in pyroptosis include the activation of caspase-3, caspase-8 and caspase-9 and the cleavage of gasdermin E, B and C (GSDME, GSDMB, and GSDMC, respectively). GSDMC is cleaved by caspase-8 and transcriptionally upregulated under hypoxic conditions through the interaction of pSTAT3 with programmed death-ligand 1. The amino-terminal PFD of gasdermin N then interacts with the plasma membrane, and 16 monomers oligomerize to form a gasdermin pore. The diameter of these pores is estimated to be in the range of 10–15 nm, which is large enough to release small proteins, including mature IL-1β (4.5 nm diameter), probably at a slow rate. Furthermore, sodium enters the cell, bringing water into the cell, which causes the cell volume to increase. This process can rapidly exceed the capacity of the membrane, resulting in membrane rupture. In response to membrane rupture, all the remaining soluble cytosolic contents are released so rapidly that it is essentially instantaneous, resulting in pyroptosis. Abbreviations: dsDNA, double-stranded DNA; PAMPs, pathogen-associated molecular patterns; DAMPs, damage-associated molecular patterns; NLRP1/3/4, NLR family pyrin domain-containing 1/3/4; AIM2, absent in melanoma 2; ER, endoplasmic reticulum; LPS, lipopolysaccharide; GSDM B/C/D/E, gasdermin B/C/D/E; IRAK-1/4, interleukin receptor associated kinase 1/4; TRAF-6, tumor necrosis factor receptor-associated factor 6; IL-1β, interleukin-1β; MCP-1, monocyte chemoattractant protein-1; ICAM-1, intercellular cell adhesion molecule-1; VCAM-1, vascular cell adhesion molecule-1; pSTAT3, phospho-signal transducer and activator of transcription 3

**Fig. 10:**
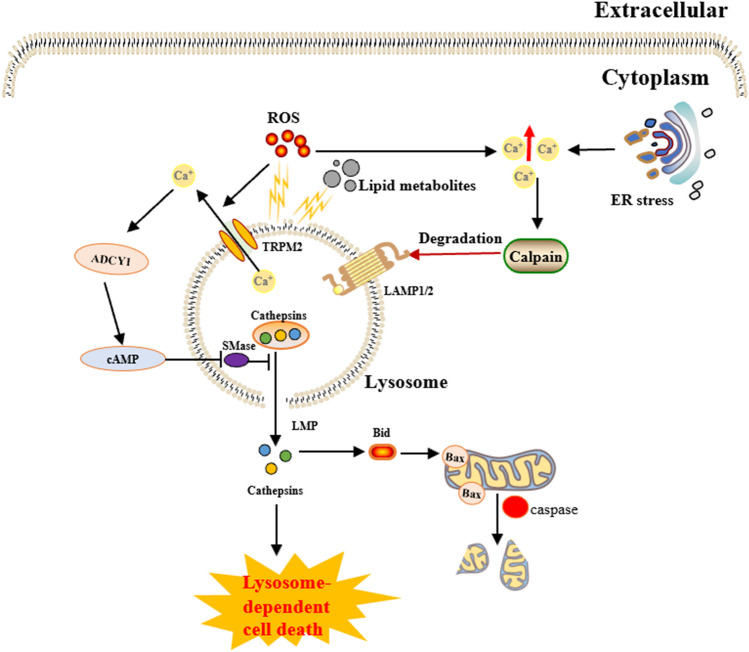
The core molecular mechanisms of lysosome-dependent cell death. Lysosome-dependent cell death is triggered by ROS or other stimuli. A surge of ROS is one of the main triggers of the increase in calcium, which can occur through hyperactivation of TRPM2 and calcium efflux from lysosomes, leading to LMP and the release of cathepsins into the cytosol. Cathepsins catalyze the formation of multiple substrates, including Bid and apoptotic proteins, and initiate caspase-dependent cell death. Lysosome-dependent cell death occurs through a process involving Ca^2+−^dependent ADCY1, followed by an increase in cAMP and ultimately the inhibition of lysosomal acid SMase. In addition, ER stress can induce an increase in cytosolic Ca^2+^. High cytosolic calcium stimulates the activation of calpain, leading to the degradation of lysosomal membrane proteins such as LAMP1/2, which causes lysosomes to rupture, resulting in lysosome-dependent cell death. Abbreviations: TRPM2, transient receptor potential melastatin 2; LMP, lysosomal membrane permeabilization; cAMP, cyclic adenosine monophosphate; ADCY1, adenylate cyclase 1; SMase, sphingomyelinase; ER, endoplasmic reticulum; LAMP1/2, lysosome-associated membrane protein 1/2; Bid, BH3-interacting domain death agonist

Consequently, a comprehensive understanding of the interplay between disulfidptosis and traditional cell death modes is critical for unraveling the complexity and diversity of cell death mechanisms.

## The mechanism of disulfidptosis

The fate and function of cells are influenced by environmental and genetic factors. One of the most critical factors that determines cell fate is redox homeostasis. Oxidative stress can produce reactive oxygen species (ROS). Cells experiencing excessive ROS must synthesize protective molecules such as glutathione to mitigate damage [62]. Glutathione synthesis requires cysteine [63], which is typically sourced from the extracellular environment via the cystine/glutamate antiporter (System XC-), which imports cystine [64]. System XC-, which is a Na^+^-dependent amino acid antiporter embedded in the phospholipid bilayer of cells, is composed of a heterodimer of the light chain SLC7A11 and the heavy chain solute carrier family 3 member 2 (SLC3A2) [65]. Concurrent with the uptake of one cystine molecule, one glutamate molecule is exported. Blocking cystine uptake triggers ferroptosis, an iron-dependent form of cell death characterized by phospholipid peroxidation, especially the peroxidation of polyunsaturated fatty acids, resulting in widespread plasma membrane abnormalities.

The maintenance of proper redox homeostasis is critical for cell survival [66]. During oxidative stress, the body protects cells by upregulating SLC7A11, facilitating the uptake of substantial amounts of cystine [67]. Within the cell, cystine, which has low solubility, is initially reduced to highly soluble cysteine, which involves NADPH [16, 28]. Subsequently, gamma-glutamylcysteine synthetase and glutathione synthetase catalyze the synthesis of glutathione, eliminating excessive ROS and reducing oxidative stress [68, 69]. Glutathione is pivotal for preventing oxidative stress, mitigating lipid peroxidation reactions, and protecting cells [63]. This process requires an adequate glucose supply, enabling sufficient NADPH production via the pentose phosphate pathway (PPP) for the timely reduction of insoluble cystine to soluble cysteine, which is crucial for glutathione biosynthesis.

### Glucose starvation and NADPH depletion

Glucose starvation, a condition in which cells experience a scarcity of glucose, disrupts the equilibrium of cellular metabolism with far-reaching consequences. This state not only affects energy production through glycolysis but also has profound implications for anabolic processes, redox balance [70], and antioxidant defense mechanisms, primarily due to its impact on the PPP [71]. The PPP, is a vital metabolic pathway that branches off from glycolysis. It plays a critical role in multiple cellular functions beyond energy generation, including the synthesis of nucleotides, pentoses for nucleic acids, and the provision of reducing equivalents in the form of NADPH [72].

NADPH is crucial for maintaining the cellular redox potential, essential for fatty acid synthesis, cholesterol biosynthesis, and perhaps most importantly, it serves as a cofactor for antioxidants like glutathione, which neutralize ROS and protect cells from oxidative stress [73]. The PPP serves as the primary source of NADPH in animal cells [74–77]. During glucose starvation, the PPP is inhibited, leading to a decrease in NADPH production and rapid depletion. Reduced NADPH levels compromise the ability of cells to regenerate reduced GSH from its oxidized form (GSSG), thereby weakening the cellular antioxidant defense system [78]. This can lead to an accumulation of ROS, causing oxidative damage to lipids, proteins, and DNA, ultimately compromising cell viability and function [79]. Impaired Lipid and Cholesterol Synthesis.

The balance between oxidants and antioxidants in the cell, crucial for maintaining homeostasis, is disrupted due to decreased NADPH. This redox imbalance can disturb protein function, modulate signaling pathways, and trigger programmed cell death. To cope with glucose starvation, cells may activate alternative metabolic routes to generate ATP and maintain redox balance, such as upregulated fatty acid oxidation, amino acid catabolism, or activating autophagy to recycle intracellular components [80]. However, these adaptive mechanisms may not fully compensate for the loss of PPP activity, especially regarding NADPH production, highlighting the importance of glucose as a fundamental energy and biosynthetic substrate for cellular homeostasis.

### SLC7A11 as a dual-edged sword in redox regulation

SLC7A11 plays a crucial role in cellular processes. By serving as a transmembrane protein, it regulates the balance between cystine and glutamate and is intrinsically linked to the maintenance of mitochondrial function [81]. Decreased SLC7A11 expression leads to inadequate cystine uptake, inhibiting timely ROS clearance and culminating in ferroptosis [29, 82]. Conversely, increased SLC7A11 expression results in excessive cystine absorption, which is cytotoxic and can trigger disulfidptosis [2]. Concurrently, extensive glutamate export decreases intracellular glutamate levels, reducing mitochondrial membrane potential, causing mitochondrial swelling and dissolution, and impairing mitochondrial function [83]. Consequently, SLC7A11 expression critically regulates cellular redox status and mitochondrial function, requiring precise modulation to maintain normal cellular physiology.

#### A decrease in SLC7A11 expression induces ferroptosis

Maintaining proper redox homeostasis is critical for cell survival [66]. Oxidative stress leads to the production of large amounts of ROS, which can hinder normal cell growth and differentiation or even lead to cell death [84]. Therefore, maintaining sufficient glutathione (GSH) levels to neutralize excess ROS and stabilize the intracellular environment is essential [85].

GSH synthesis depends on cysteine, which is derived from cystine and enters the cell from the extracellular milieu through SLC7A11. SLC7A11-mediated GSH synthesis is vital for the cellular antioxidant defense and the maintenance of intracellular stability [86]. Reduced SLC7A11 expression on the cell membrane leads to insufficient cystine uptake, causing a decrease in cysteine availability and, subsequently, a decrease in GSH synthesis [87–89]. This results in reduced activity of glutathione peroxidase-4 (GPX4), the only enzyme capable of efficiently reducing lipid peroxides in biological membranes, which requires glutathione as a cofactor [90, 91]. The inability to reduce lipid peroxides in a timely manner leads to the oxidation of intracellular Fe^2+^, which generates large amounts of lipid radicals and ROS through the Fenton reaction: Fe^2+^  + H_2_O_2_ → Fe^3+^  + (OH)^−^ + ·OH, Fe^3+^  + O^2−^ → Fe^2+^  + O_2_ [92]. Hydroxyl radicals (·OH) can attack assault polyunsaturated fatty acids embedded in the cellular membrane, instigating a self-amplifying cascade of lipid peroxidation [93]. The concentration of iron ions is meticulously governed by a cohort of iron metabolism-associated proteins, notably the iron export protein (FPN) [94], the iron regulatory hormone (Hepcidin) [95], and the iron storage molecule (Ferritin) [96].

The abundance of polyunsaturated fatty acids in the cell and plasma membranes increases susceptibility to lipid radical-induced cascade reactions, decreasing membrane thickness and compromising barrier function. ROS further accelerate damage, forming protein pores on the cell membrane and destabilizing the intracellular environment. Concurrently, lipid radicals damage the cellular lipid structure, and the resulting peroxidation products (4-hydroxy-nonenal, malondialdehyde) continue to react, perpetually damaging the cell. Ultimately, this leads to irreversible damage to the structure and function of the cell and plasma membrane, culminating in ferroptosis. SIRT1 is a NAD^+^-dependent deacetylase that plays a central role in cellular responses to metabolic stress and aging. SIRT1 has been shown to positively regulate SLC7A11 expression, thereby enhancing cystine uptake and GSH synthesis, and contributing to ferroptosis resistance [97, 98]. This finding underscores the indispensable role of SLC7A11 in cell survival.

#### Cells with high SLC7A11 expression under glucose starvation undergo disulfidptosis

SLC7A11 is recognized for its ability to neutralize excess ROS, thereby promoting cell survival under normal conditions [99]. However, research led by Professor Boyi Gan revealed that under glucose starvation conditions, SLC7A11 overexpression paradoxically leads to cell death [16]. This type of cell death has been classified as disulfide stress-induced cell death and is a novel form of programmed cell death defined by stress from disulfide bonds [2]. These findings underscore the complex role of SLC7A11 in the regulation of cellular redox homeostasis and the balance between cell survival and death.

Specifically, cystine, which is an amino acid with very low solubility, can become highly toxic when it accumulates in the cytoplasm [16, 100]. Consequently, cells with high SLC7A11 expression must rapidly convert cystine to the more soluble cysteine in the cytoplasm. This reduction is dependent on NADPH, which is produced via the PPP of glucose metabolism [30, 66]. Under glucose starvation conditions or when glucose uptake is insufficient, NADPH production via the PPP is decreased, inhibiting the prompt conversion of cystine to cysteine and potentially leading to the accumulation of abnormal disulfide bonds, such as cystine, within the cell. This abnormal accumulation can trigger disulfide stress, consequently inducing disulfidptosis [2].

Additionally, studies indicate that 2-deoxy-D-glucose (2-DG) can prevent the death of SLC7A11-overexpressing cells under glucose starvation conditions [101]. 2-DG, which is a glucose analog, inhibits glycolysis and can be diverted to the PPP to produce NADPH. This finding suggested that the protective effect of 2-DG against the death of SLC7A11-overexpressing cells under glucose-deprived conditions may involve its ability to supply NADPH for cysteine reduction.

### Formation of disulfide bonds and disulfide stress

Disulfide bonds are common chemical bonds in protein molecules and are crucial in the protein folding process [102]. These bonds significantly impact protein stability and function. Improper formation or disruption of disulfide bonds can lead to abnormal protein structure, thereby affecting protein function. In pathological conditions such as cancer or neurodegenerative diseases, abnormal disulfide bonds can result in protein dysfunction, subsequently impacting cell survival [103].

In cases of glucose starvation, NADPH depletion coupled with high SLC7A11 expression leads to increased cystine uptake but inhibits timely cystine conversion to cysteine, resulting in the accumulation of cystine and other disulfides and triggering disulfide stress [2, 16]. Activation of the Ras-related C3 botulinum toxin substrate 1 (Rac1)-WAVE regulatory complex (WRC)-actin-related protein 2/3 (Arp2/3) signaling pathway occurs, leading to abnormal disulfide bonds in actin cytoskeletal proteins. These disulfide bonds result in F-actin fiber aggregation, causing damage to the cytoskeletal structure, the loss of cell function, and ultimately cell death.

F-actin fibers are protein fibers within the cytoskeleton that provide support and facilitate movement [104]. Aggregation of these fibers damages cytoskeletal structure and leads to the loss of cell function and eventually cell death. This cell death can be mitigated by inhibiting SLC7A11 or by using reducing agents such as dithiothreitol (DTT), β-mercaptoethanol (2ME), and tris-(2-carboxyethyl)-phosphine (TCEP), which prevent disulfide stress, but not by traditional cell death inhibitors, including ferroptosis inhibitors, apoptosis inhibitors, necroptosis inhibitors, autophagy inhibitors, or ROS scavengers. This finding indicates that cell death may be mediated by SLC7A11-induced cystine accumulation and subsequent disulfide stress [2].

### F-actin contraction during disulfidptosis

Phalloidin staining was performed by Boyi Gan [2] and revealed significant morphological changes, such as cell and F-actin contraction and F-actin detachment from the plasma membrane, in SLC7A11-overexpressing cells under glucose starvation conditions. Furthermore, glucose starvation-induced actin cytoskeletal remodeling depended on SLC7A11 and could be reversed by cysteine deprivation, 2-DG, or 2ME treatment but not by treatment with ROS scavengers (Tempol or Trolox) [2]. This finding suggests that disulfidptosis is related to the formation of abnormal disulfide bonds in the cytoskeletal protein F-actin and the contraction and detachment of F-actin from the plasma membrane.

### The Rac1-WRC-Arp2/3 signaling pathway regulates disulfidptosis

Research indicates that genes and proteins such as NCK-associated protein 1 (NCKAP1), the WRC complex, and Rac1 are crucial for disulfidptosis [2, 105]. The WRC complex, which functions as a downstream effector of the small GTPase Rac, activates Arp2/3, leading to F-actin polymerization and podosome formation. NCKAP1, which is a component of the WRC complex, influences glucose starvation-induced disulfide bond formation and F-actin contraction and detachment from the plasma membrane; its deletion reduces disulfidptosis in UMRC6 cells, and its overexpression promotes disulfidptosis.

Rac1, which is a key GTPase that activates the WRC complex, enhances podosome formation and disulfidptosis in SLC7A11-overexpressing cells [2, 106]. Rac1-WRC-mediated podosome formation can promote disulfidptosis through the F-actin network in podosomes, which is a critical target for disulfide bonding between actin cytoskeletal proteins.

This finding highlights the significant role of the Rac1-WRC-Arp2/3 signaling pathway in disulfidptosis, contributes to a deeper understanding of cell death mechanisms, and suggests new targets for disease treatment.

### Other regulatory molecules and pathways related to disulfidptosis

The oxidation‒reduction status and the formation and breakage of disulfide bonds are pivotal factors that regulate disulfidptosis [107]. Various factors, including intracellular and extracellular environments and metabolic states, can affect cellular redox status, thereby influencing disulfide bond dynamics. Disulfidptosis involves sulfur oxidases and sulfatases, which impact the cellular redox state [9]. Additionally, proteins such as glyceraldehyde-3-phosphate dehydrogenase (GAPDH), thioredoxin (Trx), and peroxiredoxin (Prx) regulate disulfidptosis [108–107]. Furthermore, signaling pathways such as the NF-κB pathway [111, 112] and the JNK receptor pathway [113] are instrumental in disulfidptosis. These pathways affect disulfidptosis by regulating intracellular redox levels, protein expression and function. Since SLC7A11 is not essential in normal tissues but is highly expressed in multiple cancers, including lung [114, 115] and kidney [16, 116] cancers, it represents a promising target for novel cancer therapies. Targeting SLC7A11, glucose transport or the PPP, inducing disulfidptosis, and killing cancer cells are potential treatment strategies. Understanding the proteins and signaling pathways involved in disulfidptosis is crucial for developing cancer therapies.

## Potential application prospects of disulfidptosis

Following the discovery of disulfidptosis, extensive research has been conducted to determine its role in a range of physiological and pathological conditions. Preliminary studies suggest that disulfidptosis plays a role in the pathogenesis of diseases including cancer [2, 5, 6, 114–116], neurodegenerative diseases [7, 8], cardiovascular diseases [9], and liver diseases [10–12]. For instance, in cancer cells, altered metabolism often leads to glucose insufficiency, which can trigger disulfidptosis in cells with high SLC7A11 expression [2, 5, 6, 114–116]. Similarly, pathological changes related to disulfidptosis have been observed in neurodegenerative diseases such as Alzheimer's disease [7, 8].

Disulfidptosis has also emerged as a focal point in drug development [117]. Certain drugs can induce or inhibit disulfidptosis by modulating SLC7A11 expression or disulfide concentrations. For example, some chemical agents can target tumor cells by inducing disulfidptosis [118, 119]. Additionally, small molecule inhibitors [31] and gene therapy techniques [120] to regulate disulfidptosis are being explored.

In therapeutic contexts, inducing disulfidptosis in cancer cells can effectively eliminate these cells [121]. Modulating immune system functions can prevent autoimmune diseases [13–15]. Moreover, targeting disulfidptosis pathways may lead to treatment strategies for other diseases caused by oxidative stress imbalances, such as neurodegenerative [7, 8] and inflammatory diseases [9].

In conclusion, the study of disulfidptosis would not only advance our understanding of cell death mechanisms but also open new avenues for disease prevention and treatment.

### Potential application prospects of disulfidptosis in cancer treatment

Some studies have demonstrated that disulfidptosis is intimately related to the occurrence and development of cancer [2, 5, 6, 114–116]. Initially, the expression level of SLC7A11 in certain cancer cells was markedly higher than that in normal cells. This could be attributed to the high expression of SLC7A11, which could promote the proliferation and metastasis of cancer cells [122]. Furthermore, research indicates that high SLC7A11 expression may correlate with cancer drug resistance [123–125]. This could be due to the high expression of SLC7A11, which enhances the metabolic activity of cancer cells, thus conferring resistance to the toxic effects of certain chemotherapeutic drugs.

Research has revealed that the occurrence of disulfidptosis is closely related to the treatment and prognosis of cancers [121, 126, 127]. For instance, certain anticancer drugs might induce cancer cells to undergo disulfidptosis, thus inhibiting cancer growth and metastasis [121]. Additionally, the occurrence of disulfidptosis may be associated with cancer immune escape [126].

In conclusion, disulfidptosis is fundamentally related to the occurrence and development of cancer, and research on disulfidptosis in cancer treatment holds significant importance and value. By conducting comprehensive research on the regulatory mechanisms and applications of disulfidptosis, it is possible to generate new ideas and methods for cancer treatment.

### Potential application prospects of disulfidptosis in the treatment of neurodegenerative diseases

Recent studies indicate that disulfidptosis is also related to neurodegenerative diseases [7, 8]. In certain neurodegenerative diseases, the expression level of SLC7A11 is markedly higher than that in normal cells [118]. This could be attributed to the high expression of SLC7A11, which enhances the metabolic activity of neurons, leading to resistance to certain forms of damage; further research shows that high expression of SLC7A11 may also promote neuronal apoptosis and necrosis [128], contributing to the occurrence and development of neurodegenerative diseases.

Research has revealed that the occurrence of disulfidptosis is related to the treatment and prognosis of neurodegenerative diseases [9]. For instance, certain drugs can inhibit the progression of neurodegenerative diseases by inducing neuronal disulfidptosis. Additionally, research has suggested that the occurrence of disulfidptosis may be linked to immune escape mechanisms in neurodegenerative diseases [7].

Alzheimer's disease, which is a prevalent neurodegenerative condition, has a complex pathogenesis that has not been fully elucidated. Recent studies suggest that disulfidptosis may play a role in the occurrence and development of Alzheimer's disease [7, 8, 32]. Furthermore, a study revealed that high SLC7A11 expression could contribute to neuronal death [129], thus influencing the occurrence and development of Alzheimer's disease. Moreover, evidence indicates that the occurrence of disulfidptosis is related to the treatment and prognosis of Alzheimer's disease [7].

Research on targeting disulfidptosis to treat neurodegenerative diseases has emerged as a pivotal area in neuroscience. Comprehensive investigations of the regulatory mechanisms and applications of disulfidptosis could yield new insights and approaches for treating neurodegenerative diseases.

### Potential application prospects of disulfidptosis in cardiovascular disease treatment

The application prospects of disulfidptosis in treating cardiovascular diseases have garnered considerable attention. Recent studies have indicated that the SLC7A11 protein is intimately linked to cardiovascular diseases such as myocardial ischemia‒reperfusion [130, 131], myocardial infarction [132, 133], and myocardial hypertrophy [134] and that its association with disulfidptosis is significant. Consequently, further investigations are needed to determine the relationships between disulfidptosis and cardiovascular diseases, such as myocardial ischemia‒reperfusion, myocardial infarction, and myocardial hypertrophy.

In conclusion, the application prospects of disulfidptosis in treating cardiovascular diseases hold substantial importance and value. Comprehensive investigations of the regulatory mechanisms and applications of disulfidptosis could yield new insights and approaches for treating cardiovascular diseases.

### Potential application prospects of disulfidptosis in other diseases

In addition to its role in tumors, neurodegenerative diseases, and cardiovascular diseases, disulfidptosis has also been linked to other conditions, such as diabetes [135] and autoimmune diseases [13]. The mechanism of disulfidptosis is incompletely understood, and further research on its regulatory mechanisms and applications could lead to the identification of new approaches for treating diabetes and autoimmune diseases.

## The prospects and outlook of targeting disulfidptosis

The role and significance of disulfidptosis, which is a novel form of programmed cell death, in various diseases has been the subject of intensive study and exploration. There are several future research directions and application prospects for targeting disulfidptosis.

### In-depth study of the mechanism and regulatory network of disulfidptosis

Under conditions of limited NADPH availability due to glucose deprivation, cells with elevated SLC7A11 expression face a serious challenge. Their heightened cystine uptake leads to dual consequences: the depletion of intracellular NADPH reserves and an excessive accumulation of cystine alongside other disulfide-containing molecules. This surplus disrupts normalcy by instigating aberrant disulfide bond formation within actin cytoskeleton proteins, ultimately causing the collapse of the actin network and a state referred to as disulfidptosis [2–4].

The process is also tightly controlled by molecules like GAPDH, Trx, and Prx [108–110], and pathways such as NF-κB [111, 112] and JNK [113], which regulate redox levels, protein synthesis, and function. These interactions reveal a complex regulatory network underlying disulfidptosis, with profound impacts on cellular health and disease susceptibility.

Presently, many factors that are involved in the mechanism of disulfidptosis have yet to be identified, requiring further research to elucidate the specific molecular mechanism and regulatory network.

### Comparison of disulfidptosis with other forms of cell death

Disulfidptosis, a unique form of cell stress and death characterized by the abnormal accumulation of disulfide-bonded proteins and actin cytoskeleton collapse, stands apart from more conventionally recognized modes of cell death such as apoptosis, necrosis, autophagy, and ferroptosis. Here's a comparison highlighting the distinctive features of disulfidptosis against these other cell death processes:

#### Apoptosis

Often described as programmed cell death, apoptosis is a regulated process marked by cell shrinkage, membrane blebbing, chromatin condensation, and DNA fragmentation [136]. It plays a crucial role in development, tissue homeostasis, and immune function. Unlike disulfidptosis, apoptosis does not typically involve oxidative stress-induced protein aggregation or actin network dysfunction as central features.

#### Necrosis

Necrosis is an uncontrolled and accidental cell death usually triggered by severe physical or chemical insults. It is characterized by swelling of organelles, plasma membrane rupture, and inflammation due to cellular content release [137]. While both necrosis and disulfidptosis can be induced by oxidative stress, necrosis lacks the specific disulfide bond abnormalities and actin cytoskeleton collapse seen in disulfidptosis.

#### Autophagy

Autophagy is a lysosome-dependent degradation process that cells use to recycle damaged organelles and long-lived proteins [138]. It serves as a survival mechanism during starvation but can also contribute to cell death when overly activated or dysregulated. Unlike disulfidptosis, autophagy involves vesicular sequestration of cytoplasmic components rather than direct protein misfolding and aggregation due to disulfide bond anomalies.

#### Ferroptosis

Ferroptosis is a form of regulated cell death driven by iron-dependent lipid peroxidation. It is characterized by the accumulation of toxic lipid ROS (reactive oxygen species) and membrane damage [139]. Although both ferroptosis and disulfidptosis involve oxidative stress, they differ in that ferroptosis specifically targets lipids, whereas disulfidptosis centers around protein misfolding and aggregation due to disulfide bond imbalances.

In summary, while all these forms of cell death share some common elements like oxidative stress responses, each has distinct mechanisms and hallmarks. Disulfidptosis, with its focus on abnormal disulfide bond formation and cytoskeletal collapse, offers a unique perspective on how disruptions in protein homeostasis and redox balance can lead to cell demise, diverging from the pathways of more classical cell death modalities. Understanding these differences is crucial for developing targeted therapeutic interventions for various diseases where dysregulated cell death plays a significant role.

### Prospects for targeting disulfidptosis in disease treatment

Targeting disulfidptosis for disease treatment holds significant promise given its involvement in a range of pathologies, including neurodegenerative disorders, cancer, and aging. Here are some prospective strategies and areas of focus for therapeutic intervention:

#### Modulating SLC7A11 expression or activity

Since high SLC7A11 expression contributes to cystine uptake and subsequent NADPH depletion, therapeutics that downregulate SLC7A11 could help mitigate disulfidptosis. Small molecule inhibitors or RNA-based therapies could be explored to suppress SLC7A11 function.

#### Enhancing intracellular redox balance

Strategies to increase NADPH levels or improve the efficiency of antioxidant systems, such as boosting the activity of enzymes like glutathione reductase or NADPH-producing enzymes, could counteract oxidative stress and disulfide bond imbalances.

#### Disulfide bond modifiers

Developing compounds that selectively break abnormal disulfide bonds or enhance the activity of enzymes involved in disulfide bond formation and reduction (thioredoxin, glutaredoxins) could help restore protein homeostasis and prevent cytoskeletal collapse.

#### Actin cytoskeleton stabilizers

Agents that stabilize the actin cytoskeleton could potentially counteract the effects of disulfidptosis on actin dynamics, preserving cellular integrity and function.

#### Targeting signaling pathways

Modulating signaling pathways like NF-κB and JNK, which influence intracellular redox status and protein homeostasis, could offer a systemic approach to managing disulfidptosis. Small molecule inhibitors or activators of these pathways could be developed for therapeutic use.

#### Autophagy inducers

Given the role of autophagy in protein quality control, inducing autophagic flux could help clear abnormal disulfide-bonded proteins and mitigate the downstream effects of disulfidptosis.

Research into these areas is still nascent, but the growing recognition of disulfidptosis's importance in disease etiology underscores the potential for innovative therapeutic strategies. By studying and exploring the mechanism of disulfidptosis, a deeper understanding of its mechanism and influencing factors can be achieved, thus yielding insights for new treatment methodologies.

### Prospects for targeting disulfidptosis in drug development

Targeting disulfidptosis in drug development is an emerging area of research with promising implications for several diseases where disruptions in protein disulfide homeostasis play a key role. Here are some prospects and challenges for incorporating disulfidptosis targets into the drug development pipeline:

#### Discovery of novel targets

Identifying specific enzymes, transporters, or signaling molecules involved in disulfide bond formation, reduction, or regulation can lead to new drug targets. For example, inhibitors of cystine-glutamate antiporter (xCT/SLC7A11) have gained attention for their potential in modulating oxidative stress in cancer and neurodegeneration.

#### Small molecule therapeutics

Developing small molecules capable of modulating the activity of target proteins related to disulfide metabolism, such as glutathione peroxidases, thioredoxins, or protein disulfide isomerases (PDIs), could correct imbalances in redox state.

#### Biologicals and protein therapeutics

Monoclonal antibodies or recombinant proteins designed to bind and modulate the activity of specific proteins in the disulfide metabolism pathway may offer targeted interventions with fewer off-target effects.

#### Repurposing existing drugs

Investigating whether drugs already approved for other indications can also modulate disulfide homeostasis could accelerate the development process. For instance, some chemotherapeutic agents and antioxidants may have unexplored effects on redox balance.

In-depth investigations of the molecular mechanisms and regulatory network involved in disulfidptosis may lead to the discovery of new targets and drugs, offering novel ideas and directions for drug development to treat diseases.

## Conclusion

In conclusion, disulfidptosis is a crucial mode of cell death and is highly important for understanding biology. Through in-depth study of the molecular mechanism and regulatory network of disulfidptosis, we can reveal the mechanisms of various physiological and pathological processes in the body, provide new ideas and methods for disease prevention and treatment, offer a new theoretical basis for the development of life sciences, and make greater contributions to human health. Moreover, the study of disulfidptosis is highly important for the development of cell biology. This research can not only propel the field forward but also offer fresh perspectives and directions for the study of other forms of cell death.

## Data Availability

No datasets were generated or analysed during the current study.
